# Phenotypic traits of mesenchymal stem cell sheets fabricated by temperature-responsive cell culture plate: structural characteristics of MSC sheets

**DOI:** 10.1186/s13287-019-1431-6

**Published:** 2019-11-28

**Authors:** Mitsuyoshi Nakao, Kyungsook Kim, Kenichi Nagase, David W. Grainger, Hideko Kanazawa, Teruo Okano

**Affiliations:** 1 Cell Sheet Tissue Engineering Center (CSTEC), Department of Pharmaceutics and Pharmaceutical Chemistry, University of Utah, Health Sciences, 30 South 2000 East, Salt Lake City, UT 84112 USA; 2 Faculty of Pharmacy, Keio University, 1-5-30 Shibakoen, Minato-ku, Tokyo, 105-8512 Japan; 3 Institute of Advanced Biomedical Engineering and Science, Tokyo Women’s Medical University, 8-1 Kawada-cho, Shinjuku-ku, Tokyo, 162-8666 Japan

## Abstract

**Background:**

In most stem cell therapy strategies reported to date, stem cells are introduced to damaged tissue sites to repair and regenerate the original tissue structure and function. MSC therapeutic efficacies are inconsistent, largely attributed to transplanted MSC difficulties both in engrafting at tissue sites and in retaining their therapeutic functions from suspension formulations. MSC functional components, including cell adhesion and cell–cell junction proteins, and ECM that contribute to essential cellular therapeutic effects, are damaged or removed by proteolytic enzymes used in stem cell harvesting strategies from culture. To overcome these limitations, methods to harvest and transplant cells without disrupting critical stem cell functions are required. Cell sheet technology, exploiting temperature-responsive cell culture surfaces, permits cell harvest without cell protein damage. This study is focused on phenotypic traits of MSC sheets structurally and functionally to understand therapeutic benefits of cell sheets.

**Methods/results:**

This study verified cleaved cellular proteins (vinculin, fibronectin, laminin, integrin β-1, and connexin 43) and increased apoptotic cell death produced under standard trypsin harvesting treatment in a time-dependent manner. However, MSC sheets produced without trypsin using only temperature-controlled sheet harvest from culture plastic exhibited intact cellular structures. Also, MSCs harvested using enzymatic treatment (i.e., chemical disruption) showed higher pYAP expression compared to MSC sheets.

**Conclusion:**

Retention of cellular structures such as ECM, cell–cell junctions, and cell–ECM junctions is correlated with human umbilical cord mesenchymal stem cell (hUC-MSC) survival after detachment from cell culture surfaces. Retaining these proteins intact in MSC cultures using cell sheet technology is proposed to enhance stem cell survival and their function in stem cell-based therapy.

## Background

Cell-based therapy promises to improve current limitations of small molecule and biological drugs in regenerative medicine, exploiting rapid advances in stem cell sourcing, including embryonic stem cells (ES cells), induced pluripotent stem cell (iPS cells), and mesenchymal stem cells (MSC). In fact, over 6500 clinical trials using stem cells have been conducted worldwide (https://clinicaltrials.gov/). However, contrary to supporting preclinical animal studies, clinical studies to date show minimal or only transient improvements in therapeutic effects [1]. This non-predictive translational problem remains unelucidated. To improve the required clinical translational impact, stem cells used as biological therapy must be better understood to enhance their safety and therapeutic effects in human use.

In conventional stem cell therapies, cells isolated from donor sources (allogeneic) or patients (autologous) are expanded and cultured on plastic cell cultureware using various strategies. Cells are ultimately harvested from these single-use plastic surfaces for therapeutic use [2]. Since cultured cells generally attach to cell culture dishes strongly using intrinsic adhesion proteins (e.g., extracellular matrix and cell membrane receptors), these adhesive proteins must be released to harvest cells from culture surfaces. Two general methods are used to separate adherent cultured cells from cell cultureware: chemical and physical disruption. Chemical disruption of cell adhesive proteins is the most commonly used method in stem cell sourcing for therapy. Proteolytic enzymes (e.g., trypsin and collagenase) are added to cell culture media and general non-specific enzymatic digestion cleaves myriad proteins both on cell membrane surfaces and deposited on plasticware surfaces (e.g., culture medium-resident and cell-sourced matricellular proteins) non-specifically [3]. This uncontrolled proteolytic disruption compromises various important cell functions (e.g., cell proliferation, adhesion, survival, and migration) [4]. Significantly, the resulting harvested cell product is a single cell suspension where endogenous cell–cell associations common to tissue formation and engraftment are disrupted. Another method uses ethylenediaminetetraacetic acid (EDTA) as a calcium chelator to remove calcium ions from integrins and calcium-obligate cell binding proteins, releasing cells without exogenous enzymatic action. This method however suffers from EDTA cell toxicity [5].

By contrast, cell harvesting using physical disruption manually and mechanically shears adherent cultured cells from cell cultureware surfaces using a cell scraper. Ubiquitous protein cleavage occurring during chemical (enzymatic) disruption of cell cultures is not observed in physical disruption: cell proteins are spared. However, physical disruption harvesting methods are not used in cell therapy because harvested cells form heterogeneous aggregated clusters [6]. Therefore, reproducible homogeneous cell products required for treatment are difficult. Physical disruption is used generally for cell proteomic analyses. These features of conventional cell harvesting methods using either chemical or physical cell disruption limit current clinical applications for stem cells.

To improve cell harvest from cell culture surfaces, Okano et al. have extensively reported cell sheet technology to harvest cultured cells using small changes of temperature without enzymatic treatment or cell or protein disruption [7]. This cell sheet technology uses unique cell cultureware modified with thin grafted layers of temperature-responsive polymer, poly *N*-isopropylacrylamide (PIPAAm) [8]. PIPAAm is well-known to exhibit an aqueous lower critical solution temperature (LCST) at 32 °C. Temperature-responsive cell culture dishes (TRCD) change rapidly from hydrophobic to hydrophilic as cell culture surface temparture is reduced below 32 °C. Using this approach, adherent cultured cells on TRCD are harvested without any enzyme treatment as a contiguous intact viable cell sheet. Aqueous media penetrate spontaneously into the PIPAAm polymer interface between adherent cell and TRCD at temperatures below 32 °C, expanding PIPAAm chains under hydration and physically separating cell surfaces from TRCD surfaces. This cell sheet technology represents a unique method to harvest cells gently and non-disruptively, enabling harvest of adherent cells from TRCD without damage to ECM, cell surface proteins, cell receptors, or intercellular proteins important to cell survival and function. Furthermore, recently, several allogeneic cell sheet therapies have also reported using MSC sheets in wound healing, heart, and pancreas regeneration [9–11].

Given these important advantages, cell sheet technology can facilitate improvements in stem cell cultures for cell therapy currently limited by chemical disruption harvesting and resulting single cell suspensions used for injection. The study aimed to clarify some basic scientific cell harvesting advantages of MSC sheet technology, extending the autologous primary cell sourcing for sheets currently used to treat several human diseases [12–18]. MSCs recovered as sheets using cell sheet culture technology were structurally and functionally compared to cells harvested using both chemical and physical disruption methods.

## Materials and methods

### Antibodies

The following primary antibodies were used for cell immunostaining: CD 44 (ab6124) (Abcam, Cambridge, USA), actin (ab8226) (Abcam), vinculin (ab129002) (Abcam), fibronectin (ab6328) (Abcam), laminin (ab11575) (Abcam), integrin β-1 (ab179471) (Abcam), connexin 43/GJA1 (ab11370) (Abcam), yes-associated protein (YAP) (#140794) (Cell Signaling Technology (CST), USA), phospho-YAP (Ser127, #4911)) (CST), and GAPDH (ab9484) (Abcam). Alexa Fluor 568 goat anti-rabbit, 568 goat anti-mouse, 488 goat anti-rabbit, and 488 goat anti-mouse secondary antibodies (Life Technologies, Carlsbad, USA) and HRP-conjugated goat anti-mouse and goat anti-rabbit (Abcam) were also used as described below.

### Human umbilical cord-derived mesenchymal stem cell (hUC-MSC) culture

Banked hUC-MSCs isolated from the subepithelial layer of human umbilical cord tissue (Jadi Cell LLC, Miami, USA) were cultured in Dulbecco’s modified Eagle’s medium (DMEM, Gibco, USA) supplemented with 10% fetal bovine serum (FBS) (Gibco), 1% GlutaMAX (Gibco), 1% MEM non-essential amino acids (NEAA) (Gibco), 100 units/mL penicillin, and 100 μg/mL streptomycin (Gibco) [19]. hUC-MSC was incubated at 37 °C with 5% CO_2_ in a humidified chamber and passaged when cells reached confluence. hUC-MSCs passaged with TrypLE (Gibco) treatment for 5 min were sub-cultured in media at 3000 cells/cm^2^ between passages 4 and 6.

### Preparation of hUC-MSC sheets, and chemical and physical harvesting of MSCs

**Fig. 1:**
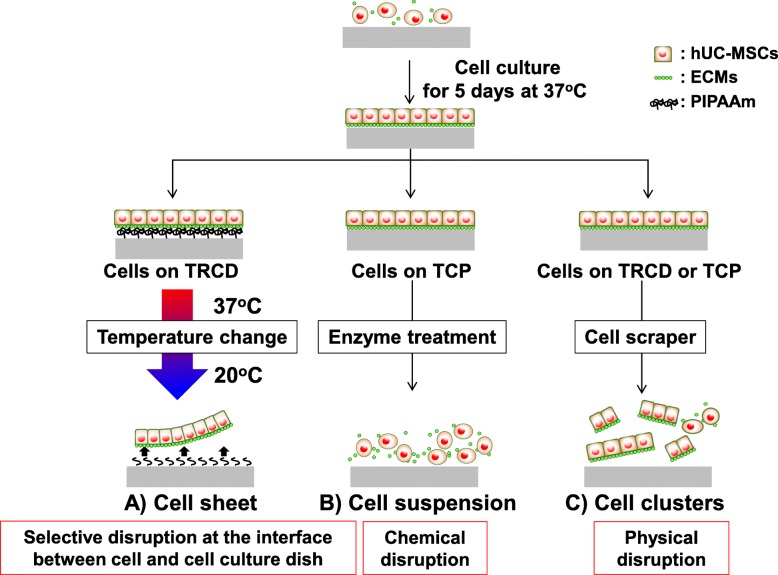
Illustration of cell harvesting process. hUC-MSCs were seeded on a 35-mm-diameter TRCD or TCP and cultured for 4–5 days to reach confluence. hUC-MSC was harvested using three different methods: cell sheet technology, chemical disruption, and physical disruption. (A) Cell sheet harvested from TRCD by temperature change. (B) Cells were treated by enzyme (trypsin) on TCP. (C) Cells were harvested using cell scraper from TCP

hUC-MSCs were seeded on 35-mm diameter TRCDs (CellSeed, Tokyo, Japan) at a density of 2 × 10^5^ cells/dish (day 0) and cultured to confluence (days 4–5). Cell culture media including 16.4 μg/mL of ascorbic acid (Wako, Osaka, Japan) were replaced at 1 day after seeding. hUC-MSC cultures were harvested at 4–5 days after seeding as intact monolayer sheets from TRCD within 60 min by reducing culture temperature to 20 °C (Fig. 1). Morphological changes and cell growth rates of hUC-MSC were assessed for 4 days. To count the total cell number (cell growth rate) on 35-mm-diameter tissue culture polystyrene (TCP) and TRCD, hUC-MSCs were dissociated with TryPLE and were counted using the trypan blue (Gibco) exclusion test using a hemocytometer at 24, 48, 72, and 96 h after seeding. To prepare chemical and physical disrupted cells, hUC-MSC was seeded on TCP (Thermo Fisher Scientific, USA) at a density of 2 × 10^5^ cells/dish and culture under conditions identical to cell sheet preparation. At days 4–5, hUC-MSC cultures were harvested as cell suspensions from TCP by 0.05% or 0.5% trypsin-EDTA (Gibco) (chemical disruption) or cell scraper (Thermo Fisher Scientific, USA) (physical disruption) (Fig. 1).

### Hematoxylin and eosin (H&E) staining of hUC-MSC sheets

Cell sheet samples were fixed with 4% buffered paraformaldehyde (PFA), embedded in paraffin, and cut into 4-μm-thick sections. These sections were stained with Mayer’s hematoxylin and 1% eosin alcohol solution and mounted with Permount™ (Thermo Fisher Scientific, USA). Stained samples were visualized using a BX53 microscope (Olympus, Tokyo, Japan).

### Morphological observation of hUC-MSCs using scanning and transmission electron microscopy

For scanning electron microscopy (SEM) analysis, samples were rinsed in wash buffer (0.1 M sodium cacodylate buffer with 2.4% sucrose and 8 mM calcium chloride) for 5 min and then fixed with 2% osmium tetroxide (OsO_4_) in wash buffer for 1 h at room temperature. Samples were rinsed with DI water to remove unbound osmium stain, then dehydrated through a graded ethanol series. Subsequently, ethanol was replaced with hexamethyldisilazane (HMDS) and dried at − 30 °C. Dry samples were observed under SEM (FEI Quanta 600 FEG, FEI, Hillsboro, USA). For transmission electron microscope (TEM) analysis, samples were fixed with a mixture of 2% PFA, 2% glutaraldehyde, and 2% OsO_4_ in 0.1 M sodium phosphate buffer and dehydrated in a graded ethanol series. Samples were then embedded in epoxy resin and cut to slices of 70-nm thickness. These ultrathin sections were observed by TEM (JEOL JEM-1400 Plus, JEOL, Tokyo, Japan).

### Cell viability assay

Cell viability was measured using a live–dead viability/cytotoxicity assay (Thermo Fisher Scientific, USA). MSC sheets and trypsin-treated MSC groups were washed twice with phosphate-buffered saline (PBS) and incubated with live–dead working solution (2 mM calcein AM and 4 mM ethidium homodimer-1) for 30 min at 37 °C in the dark. These samples were washed with 1× PBS, visualized using an AX10 microscope (Carl Zeiss Microimaging, Göttingen, Germany), and analyzed with Axiovision software (Carl Zeiss Microimaging) (Ex/Em 517 nm/617 nm ethidium homodimer-1; Ex/Em 494 nm/517 nm calcein). Numbers of live and dead cells in single suspension and MSC cell sheet groups were counted using ImageJ (National Institutes of Health), whereas live cells in cell sheets were calculated based on the following:

$$ \mathrm{Number}\ \mathrm{of}\ \mathrm{live}\ \mathrm{cell}\mathrm{s}\ \mathrm{in}\ 1\ \mathrm{picture}=\frac{\mathrm{Area}\ \mathrm{of}\ 1\ \mathrm{picture}\ \left({\mathrm{cm}}^2\right)\ }{\mathrm{Total}\ \mathrm{area}\ \mathrm{of}\ \mathrm{cell}\ \mathrm{sheet}\ \left({\mathrm{cm}}^2\right)}\times \mathrm{Total}\ \mathrm{cell}\ \mathrm{number} $$

The ratio of dead cells to live cell number was calculated to compare cell survival rate in each sample. A total of 15 pictures were taken for each group, and the average values of the ratio of dead cells to live cell number were calculated.

### Qualitative analysis of proteins important to cell

hUC-MSCs (2 × 10^5^ cells/dish) were cultured for 4–5 days and harvested by (1) temperature change (cell sheet technology), (2) trypsin treatment (chemical disruption), or (3) cell scraper (physical disruption) (Fig. 1). Cells were lysed with cell lysis buffer (RIPA buffer, proteinase inhibitor and phosphatase inhibitor) (Thermo Fisher Scientific, USA) for 15 min at 4 °C to isolate protein extracts. Samples were then sonicated three times for 9 s. Protein concentration of each sample was determined by Bradford protein assay. Samples containing identical protein amounts (10 μg) were denatured at 70 °C for 10 min and loaded onto SDS-PAGE gels (3–8% tris-acetate gels or 4–12% tris-glycine gel (Thermo Fisher Scientific, USA)) and transferred electrophoretically to polyvinylidene fluoride or polyvinylidene difluoride (PVDF) membranes (LC2002, Thermo Fisher Scientific). Membranes were treated with blocking solution (5% bovine serum albumin, BSA) for 1 h at room temperature and incubated with primary antibodies at 4 °C overnight: actin (1:1000 dilution), vinculin (1:10000 dilution), fibronectin (1:2000 dilution), laminin (1:1000 dilution), integrin β-1 (1:2000 dilution), connexin 43 (1:8000 dilution), YAP (1:1000 dilution), pYAP (Ser127) (1:1000 dilution), and GAPDH (1:5000 dilution). Incubated membranes were then treated with appropriate HRP-conjugated secondary antibodies at room temperature for 1 h. The membrane was visualized by using enhanced chemiluminescence (FluorChem HD2, ProteinSimple, California, USA). Protein expression levels were normalized to the GAPDH housekeeping gene.

### Immunocytochemistry staining of proteins related to cell functions

A hUC-MSC sheet sample was embedded in paraffin and stained for CD 44. Cultured MSC cell sheets and trypsin-treated MSCs were separately detached from cell culture dishes and immunostained immediately after cell detachment for actin, vinculin, fibronectin, laminin, collagen-1, integrin β-1, and connexin 43 stains. Cell sheets and trypsin-treated cells were fixed in 4% buffered PFA and permeabilized with 0.1% Triton X-100 (Thermo Fisher Scientific, USA). Samples were blocked with 1% BSA in 10% goat serum for 15 min and then incubated in primary antibodies (same as above) overnight at 4 °C: CD 44 (1:100 dilution), actin (5 μg/ml), vinculin (1:50 dilution), fibronectin (1:100 dilution), laminin (1:50 dilution), collagen-1 (1:100 dilution), integrin β-1 (1:200 dilution), and connexin 43 (1:100 dilution) in the presence of 1% BSA with 10% goat serum. The samples were then treated with appropriate Alexa Fluor-conjugated secondary antibodies (same as above) for 1 h, exposed to mounting solution (ProLong Gold Antifade Mountant with DAPI, Thermo Fisher Scientific, USA), and inspected using a confocal laser scanning microscope (FV1000, Olympus).

### Gene expression analysis of cell sheets

Total RNA from cell sheets was extracted using Trizol and PureLink RNA Mini Kit (Life Technologies, Carlsbad, USA) according to the manufacturer’s protocols. cDNA was prepared from 1 μg of total RNA using high-capacity cDNA reverse transcription kits (Life Technologies). qPCR analysis was performed with TaqMan Universal PCR Master Mix using an Applied Biosystems Step One instrument (Applied Biosystems™, Foster City, USA). Gene expression levels were assessed for the following genes: (1) glyceraldehyde 3-phosphate dehydrogenase (*GAPDH*; Hs99999905_m1) as a housekeeping gene, (2) hepatocyte growth factor (*HGF*; Hs00379140_m1), (3) vascular endothelial growth factor (*VEGF*; Hs99999070_m1), and (4) interleukin 10 (*IL-10*; Hs00961622_m1). All primers were manufactured by Applied Biosystems. Relative gene expression levels were quantified by the comparative CT method (*N* = 3). Gene expression levels were normalized to GAPDH expression levels.

### Statistical analysis

All values are expressed as the mean ± SEM. Two-way analysis of variance followed by Tukey’s post hoc test was used to evaluate differences between more than two groups. Probabilities (e.g., *p < 0.05 or **p < 0.01) were considered significant.

## Results

### hUC-MSC sheet preparation

**Fig. 2:**
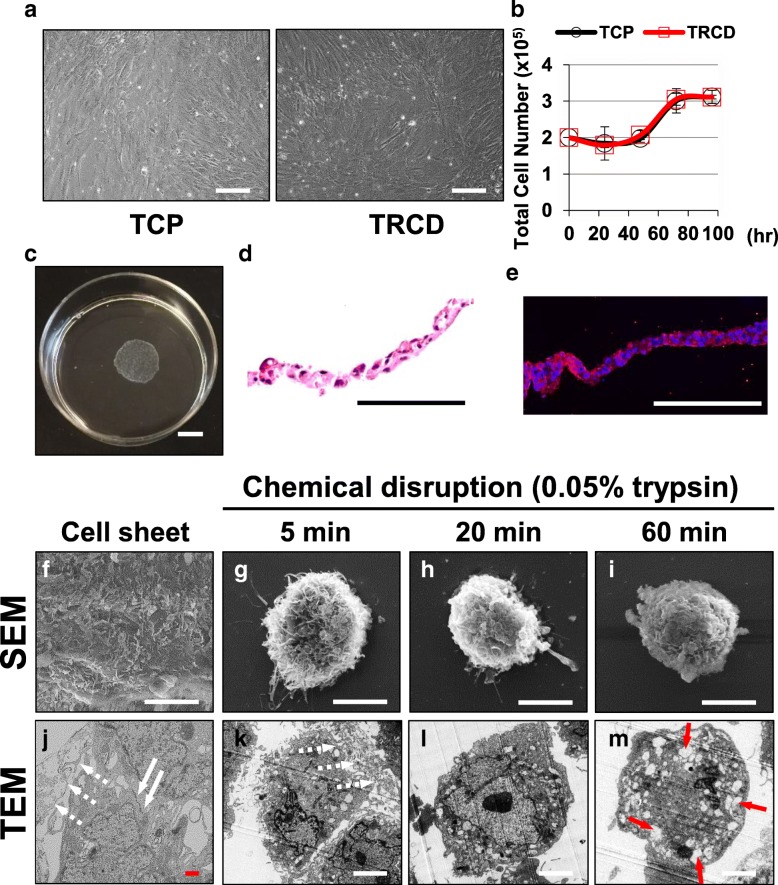
Preparation of hUC-MSC sheets. **a** Cells were cultured on conventional tissue culture plastic (TCP) or temperature-responsive cell culture dish (TRCD) for 4–5 days. **b** Cell number was counted using hemocytometer when cultured on TCP or TRCD for 100 h. **c** Cells cultured on TRCD were detached in sheet form by temperature reduction. **d**-**e** Histological analysis of cell sheets was performed by H&E stain and IHC. **f**–**i** Cell surface morphology was observed using SEM. **j**–**m** Microstructures of hUC-MSC sheets and single hUC-MSCs were analyzed using TEM. White arrows indicated cell junction, red arrows indicated ECMs and yellow arrows indicate endoplasmic reticulum in b. Scale bars indicate 200 μm (**a**, **d**, and **e**), 10 mm (**c**), and 5 μm (**j**–**m**)

Cells cultured on TRCD change morphology from rounded to spindle shape when attached to TRCD. This same morphological transition was also observed in these cells cultured on TCP (Fig. 2a). Additionally, growth rates for hUC-MSCs cultured on TRCD are similar to those on TCP (Fig. 2b). This indicates that TRCD does not affect MSC growth and morphologies. Furthermore, cells successfully detached in intact contiguous sheet form from TRCD under temperature reduction from 37 to 20 °C (Fig. 2c). Cell sheets comprise tight monolayers maintaining cell–cell binding (Fig. 2d) and maintain stem cell surface marker (CD 44) (Fig. 2e).

### hUC-MSC sheet and MSC microstructural comparisons

Surface and intercellular structures of hUC-MSC sheets observed by SEM (Fig. 2f–i) and TEM (Fig. 2j–m) exhibit connected cell membrane structures on cell surfaces, supporting hUC-MSC sheet preservation of native cell structures after cell sheet TRCD detachment (Fig. 2f). In contrast, hUC-MSCs treated with 0.05% trypsin and harvested as a cell suspension show single cell shapes with no inter-connected structures (Fig. 2g–i). In addition, cell surfaces in 0.05% trypsin-treated groups (i.e., for 5, 20, and 60 min) lost their ECM-like surface structure by trypsin treatment in a time-dependent manner (Fig. 2g–i).

Under TEM analysis, hUC-MSC sheets exhibit ECM (white dotted line) and cell–cell junctions (white solid arrow) (Fig. 2j). However, hUC-MSCs harvested with 0.05% trypsin for 5 min are absent in any cell–cell junctions and ECM compared to cell sheet groups (Fig. 2k). Furthermore, when hUC-MSCs were treated with 0.05% trypsin for 20 and 60 min, hUC-MSCs lost filopodia on cell surfaces with loss of clear nuclear morphology (Fig. 2l and m). hUC-MSCs treated with 0.05% trypsin for 60 min retain clear endoplasmic reticulum (red arrows) (Fig. 2m). SEM and TEM results together indicate that hUC-MSC sheets retain both cell surface and intercellular proteins (e.g., filopodia, ECM, and cell–cell junctions) after TRCD harvest. In contrast, all hUC-MSCs treated with 0.05% trypsin groups showed cleaved ECM and cell–cell junctions and altered nuclei. These findings suggest that trypsin treatment (chemical disruption) damages cell structures (i.e., junction proteins, ECMs, nuclei, and endoplasmic reticulum) upon chemical (enzymatic) harvest.

### hUC-MSC maintains cytoskeletal actin filament proteins related to cell dynamics

**Fig. 3:**
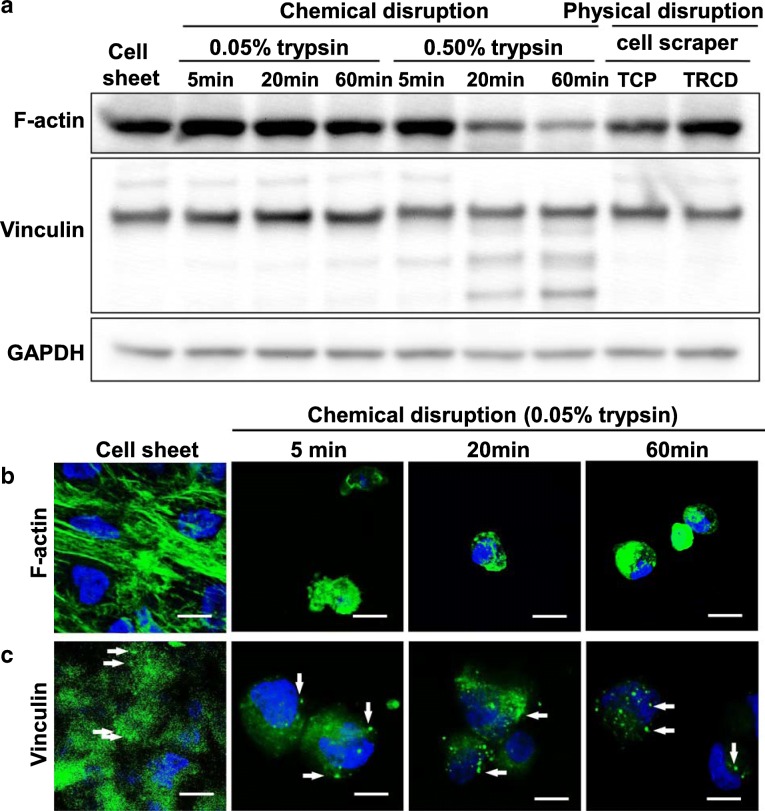
Cell dynamics-related protein expression analysis using western blot and immunohistochemistry. **a** Western blot of F-actin, vinculin, and GAPDH in whole cell lysates (10 μg protein/lane). Immunostaining of **b** F-actin (green), **c** vinculin (green), and DAPI (blue). Scale bar = 10 μm. White arrows indicate vinculin positive area

Glyceraldehyde 3-phosphate dehydrogenase (GAPDH) protein expression was used as a loading control to normalize protein amounts for western blotting assay comparisons. GAPDH protein expression level was similar across all groups. Cells treated with 0.50% trypsin for 20 and 60 min expressed lower actin levels than those in cell sheet, 0.05% trypsin, and cell scraper-harvested groups (Fig. 3a). This indicates that 0.50% trypsin cell treatment also disrupts normal actin in the cell cytoplasm. To observe cytoskeletal structure, hUC-MSCs were immunostained for actin. Cell sheet-harvested groups exhibit actin stress fibers after cell sheet harvest from TRCD (Fig. 3b). In contrast, groups treated with 0.05% trypsin for 5, 20, and 60 min showed actin-positive areas; however, distinct stress fibers are not observed (Fig. 3b). Amounts of F-actin protein were similar in cell sheet and 0.05% trypsin-treated groups (Fig. 3a). However, only cell sheet groups maintained clear actin stress fiber structures.

Vinculin expression was observed in both cell sheet and 0.05% trypsin-treated groups under immunohistochemistry staining (Fig. 3c). Degraded lower molecular weight bands in western blot analysis of vinculin expression were observed in trypsin-treated groups (Fig. 3a), indicating that vinculin in culture is cleaved by trypsin. Cells treated with trypsin showed delocalized actin fiber structures, reduced actin protein, and cleaved vinculin protein, suggesting that trypsin cleaves these proteins related to cytoskeleton and cell dynamics. This cleavage was increased when trypsin concentration was increased (Fig. 3a).

### hUC-MSC sheets maintain extracellular proteins related to cell adhesion

**Fig. 4:**
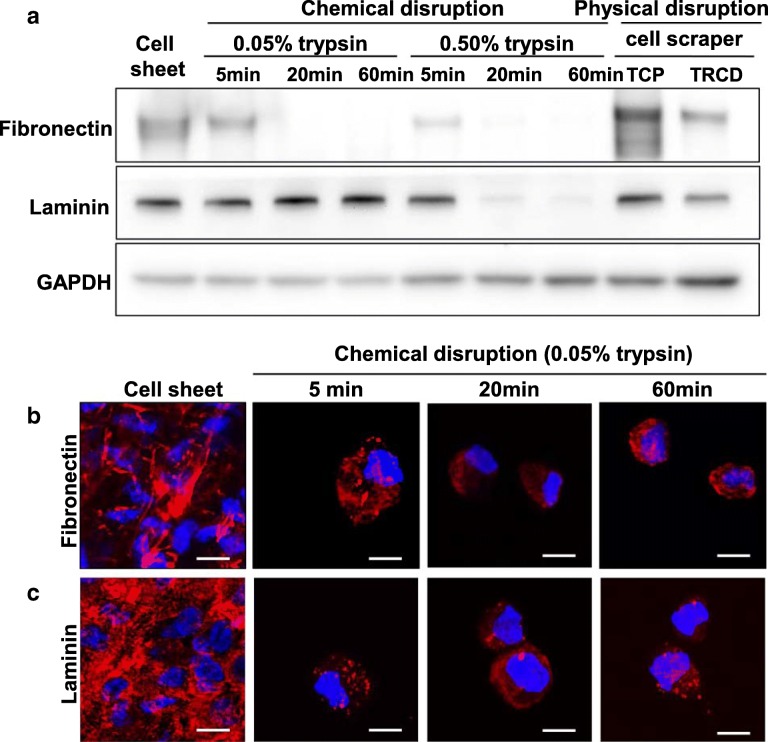
ECM protein expression analysis using western blot and immunohistochemistry. **a** Western blot of fibronectin, laminin, and GAPDH in whole cell lysates (10 μg protein/lane). Immunostaining of **b** fibronectin (red), **c** laminin (red), and DAPI (blue). Scale bar = 10 μm

Cell sheet, 0.05% or 0.50% trypsin treatment for 5 min, and cell scraper-harvested groups were qualitatively compared, given unequal loading of GAPDH control, for fibronectin expression by western blot assay. Groups from 0.05% and 0.50% trypsin treatment for 20 and 60 min exhibited no detectable fibronectin (Fig. 4a). Laminin expression was observed in cell sheet, 0.05% trypsin treatment, 0.50% trypsin treatment for 5 min, and cell scraper groups. However, 0.50% trypsin treatment groups for 20 and 60 min showed no detectable laminin expression (Fig. 4a).

Cells were immunostained with fibronectin and laminin antibodies to observe these ECM proteins (Fig. 4b and c). Higher expression of fibronectin and laminin was observed in the cell sheet group, compared to cells harvested with 0.05% trypsin (Fig. 4b and c). These results support adherent cell sheet detachment and harvest from TRCD without ECM disruption. In contrast, ECM proteins are cleaved with trypsin treatment and cell detachment from TCP (Fig. 4a).

### hUC-MSC sheets maintain cell junction proteins associated with cell–cell communication

**Fig. 5:**
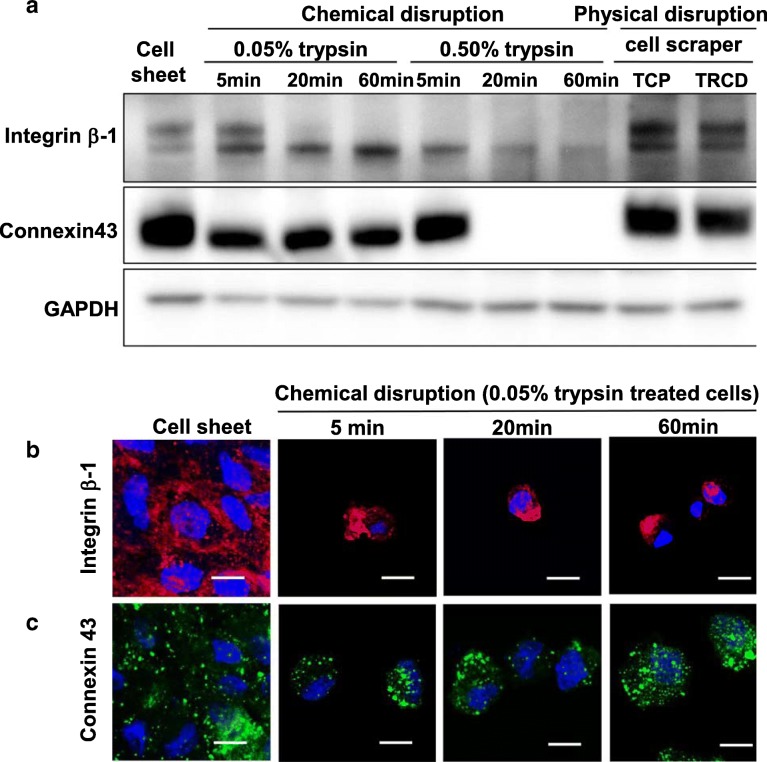
Cell–ECM and cell–cell junction protein expression analysis using western blot and immunohistochemistry. **a** Western blot of integrin β-1, connexin 43, and GAPDH in whole cell lysates (10 μg protein/lane). Immunostaining of **b** integrin β-1 (red), **c** connexin 43 (green), and DAPI (blue). Scale bar = 10 μm

Cell sheet, 0.05% trypsin treatment for 5 min, and cell scraper-harvested groups display similar integrin β-1 expression (Fig. 5a). Integrin β-1 was cleaved gradually as trypsin concentration and treatment time increase. Connexin 43 is expressed in cell sheet, 0.05% trypsin-treated (5, 20, 60 min), and 0.5% trypsin-treated (5 min) groups (Fig. 5a). However, western blotting showed 0.50% trypsin treatment for 20 and 60 min eliminated connexin 43 detection (Fig. 5a), suggesting that connexin 43 protein is cleaved by 0.50% trypsin treatment for 20 and 60 min. Structural observation of cell junction proteins was performed by immunostaining of integrin β-1 and connexin 43. Cell sheet groups showed positive expression of integrin β-1 throughout the cell sheet, whereas integrin β-1 was barely detected only sparingly on cell surfaces in 0.05% (Fig. 5b). Connexin 43 was observed in all groups (Fig. 5c) consistently over all cells in cell sheet and 0.05% groups. This suggests that cell sheets retain cell–cell junction proteins and cell–cell communication. However, trypsin treatment cleaved cell–cell adhesion proteins (i.e., single cell suspensions), suggesting trypsin treatment disrupts their cell-to-cell communication network.

### Trypsin harvesting induces cell death

**Fig. 6:**
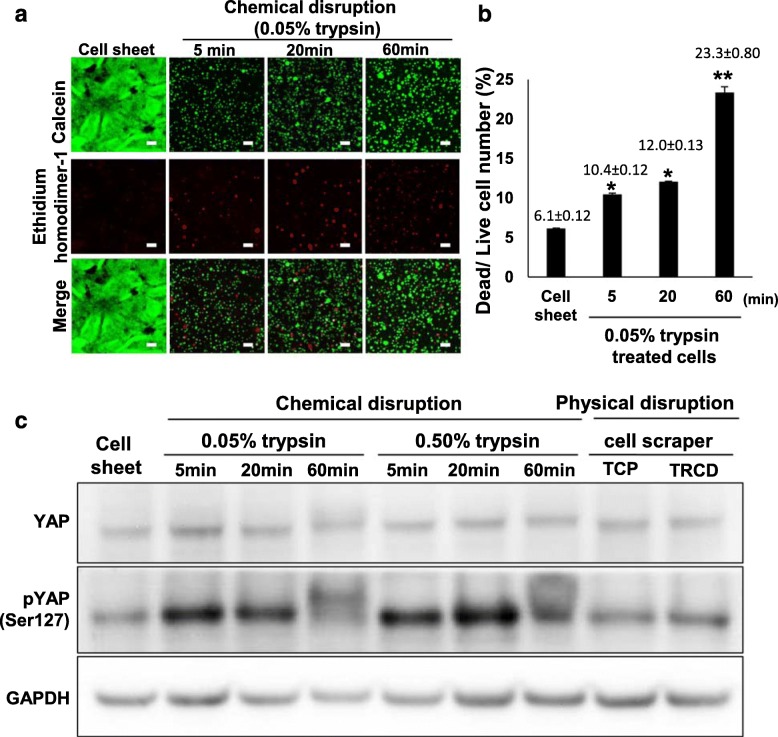
Cell viability of cell sheets. **a**, **b** Live–dead cell viability assay: **a** live (green) and dead (red) staining of cell sheet and cell suspension produced by chemical disruption. **b** Live cell and dead cell ratio was calculated using ImageJ. **c** Cell mechanosensor expression analysis of YAP proteins using western blot. YAP, pYAP, and GAPDH in whole cell lysates (10 μg protein/lane). Scale bar = 100 μm

Cells were stained with calcein and ethidium homodimer-1 immediately after cell detachment by trypsin treatment (TCP) or temperature change (TRCD cell sheet harvest). Green false color shows live cells; red color shows dead cells (Fig. 6a). The dead cell to live cell ratios in 0.05% trypsin treatment groups for 5 and 20 min were similar (Fig. 6b). The dead cell to live cell ratio in 0.05% trypsin treatment groups for 60 min significantly increased, compared to cells treated with 0.05% trypsin for 5 and 20 min (Fig. 6b). This result supports cell death induced by trypsin treatment. In addition, the dead cell to live cell ratios in cell sheet group were significantly lower compared to those in cells treated with 0.05% trypsin for 5, 20, and 60 min (Fig. 6b).

### Yes-associated protein (YAP) phosphorylation is induced by trypsin treatment

YAP and phospho-YAP (pYAP) expression in cell sheet, 0.05% and 0.50% trypsin treatment for 5, 20, and 60 min, and cell scraper-harvested groups was determined using western blotting (Fig. 6c). All groups showed similar YAP protein expression, whereas expression of pYAP was increased in 0.05% and 0.50% trypsin-treated cells compared to cell sheet and cell scraper groups (Fig. 6c). This demonstrates that trypsin treatment inhibits YAP activity and induces YAP phosphorylation.

### Paracrine factor secretion ability is enhanced in cell sheet

**Fig. 7:**
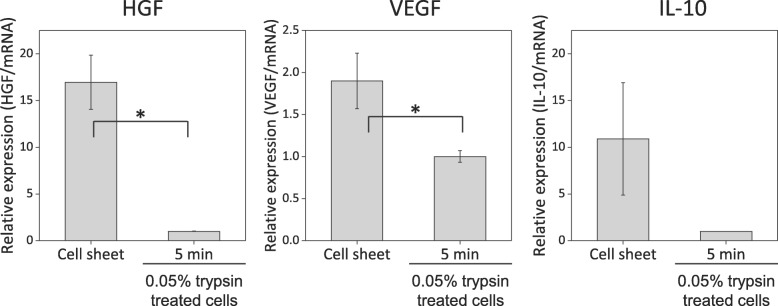
Paracrine factor secretion ability. HGF, VEGF, and IL-10 gene expression levels of cell sheet and 0.05% trypsin treatment for 5 min groups were analyzed using qPCR. *p < 0.05, *n* = 3

To verify functional differences in 0.05% trypsin treatment for 5 min and cell sheet groups, gene expression levels of secreted cytokines (HGF, VEGF, and IL-10) related to the paracrine effect of stem cells were analyzed. HGF, VEGF, and IL-10 gene expression levels in the MSC sheet group were higher than those of the 0.05% trypsin treatment for the 5 min group (Fig. 7). Particularly, the IL-10 gene expression level of 0.05% trypsin-treated cells (*n* = 2) was undetectable (Fig. 7). These results indicate that cell sheet structure enhances the paracrine secretion ability of MSCs.

## Discussion

To address culture methods and viability issues possibly affecting MSC stem cell production for therapy, the present study compares MSC structural changes after several different cell harvesting methods for human allogenic MSCs recently reported in clinical trials [20]. Stem cell cultures required to produce large quantities of cells for patient use (i.e., 10^6^–10^8^ cells/dose) are compromised by using cell-disruptive methods [21, 22]. Chemical disruption (e.g., trypsin or collagenase enzyme treatment) is widely used in stem cell culture as the basis for producing sufficient cells for therapy. However, as these chemical disruption methods non-specifically proteolyze many cell surface proteins and produce isolated single cell suspension by disrupting cell–cell connections, they are unable to preserve cell structural components or normal cell–cell communication required for normal functions. Such enzymatic treatment also disrupts extracellular matrix and intercellular proteins (via cell–cell and cell–ECM junctions). Ultimately, this harsh treatment required to harvest adherent cells from plastic compromises cell phenotypic preservation and viability and induces apoptosis [23].

MSC cell therapies are claimed to produce therapeutic anti-apoptotic and anti-fibrotic effects and regenerative and immunomodulatory properties [24]. For decades, preclinical research using animal models and clinical trials has supported stem cell therapeutic effects in treating several diseases. However, clinical results to date are inconsistent and unimpressive in most cases [1, 25]: major limitations remain to be addressed, one of which is low cell engraftment rate (less than 3% of intravenous or tissue-injected cells [26]) and low cell survival rate (cell death occurs within 2 days). Low cell engraftment rate is caused by several factors: (1) use of injected cell suspensions that have been enzymatically treated and compromised; (2) mechanical leakage of injected cells post-injection due to weak cell adhesion to targeted tissue [27]; (3) cell death, including both necrosis and apoptosis, induced by harsh inflammatory diseased tissue micro-environments; and (4) limited self-renewal capacity of injected stem cells due to compromised cell–cell communication [28]. Chemically disruptive cell harvesting methods disrupt both extracellular and intercellular proteins with functional relationships to cytoskeleton [29], cell junction, cell metabolism, and cell growth. Hence, cells harvested by chemical disruption (e.g., trypsin-treated cells) have insufficient ECM required to adhere to target tissue and insufficient cell junctions to maintain normal cellular functions through graft–host communication (Figs. 4 and 6). By contrast, hUC-MSC sheets harvested by cell sheet technology using TRCD maintain structures typical of native tissue-like inter-connected cells, including ECM components and cell junctions (Figs. 2, 4, and 5). These finding support hMSC sheet retention of cell surface and membrane proteins to improve MSC adhesion, cell–cell communication, and cellular functions [30]. In fact, previous studies showed that cardiac cell sheets consistently manifest greater cell survival on target tissue sites for 4 weeks compared to injected cell suspensions [31]. Enhanced MSC survival is suggested to result from intrinsic ECM expression retained in MSC sheets that likely enhance cell adhesion to and communication with target tissue [32]. Furthermore, intact cell–cell structures including cell junctions facilitate cell communication [33].

TEM results show that extracellular protein cleavage was observed in cells treated with 0.05% trypsin for 5 min. Cytoplasmic disturbance was observed after 20 min of 0.05% trypsin treatment, with cell nuclear changes observed at 60 min of 0.05% trypsin treatment (Fig. 2m). In addition, endoplasmic reticulum changes related to cell death [34] are observed at 60 min of 0.05% trypsin treatment (Fig. 2m). Integrins are known as a key protein to improve MSC survival and play roles in natural interactions between cell membranes and ECMs such as fibronectin and laminin [35]. Integrins are part of the cell’s outside-in signaling pathway [36] linking extracellular binding events to the cytoskeleton (actin) through adopter proteins (vinculin) and focal adhesion formation, key to cell survival, cell adhesion, and tissue repair [37]. Trypsin-induced cleavage of integrin β-1 as well as cytoskeletal F-actin, focal adhesion protein vinculin, ECM (fibronectin and laminin) is evident in Figs. 3, 4, and 5. On the other hand, hUC-MSC sheet retains intact integrin β-1, cytoskeleton, focal adhesion protein vinculin, and ECM proteins fibronectin and laminin even after TRCD detachment (see Figs. 3, 4, and 5). These findings suggest that trypsin enzymatic digestion induces cleavage of integrin β-1 proteins, which is correlated to cell adhesion and survival.

YAP is a major cell mechanosensor, localized at cell nuclei to regulate cell survival and proliferation [38]. Cell mechanosensors control cellular homeostasis by converting extracellular physical stimuli to intracellular chemical stimuli. YAP is inhibited via phosphorylation of Ser127 (phosphor-YAP, pYAP), resulting in cytoplasm retention and induction of cell death. When cells lose cell–ECM junctions, cell death, namely anoikis, is induced subsequently by YAP phosphorylation [35]. Cell death is known to be induced through YAP inhibition and subsequent pYAP induction. Similarly, breakdown of cell–ECM connections induces apoptotic cell death through inhibition of YAP [39]. Cells treated with trypsin show destroyed integrin β-1 (Fig. 5). This cleavage of integrin β-1 inactivates YAP and induces pYAP (Fig. 6). Eventually, cell death occurs in trypsin-treated cell groups. In contrast, hUC-MSC sheets maintain integrin β-1 and reduced pYAP expression (Figs. 5 and 6), showing significantly higher cell survival rates (Fig. 6). pYAP is reported to be induced not only by integrin β-1 cleavage but also by inhibition of F-actin polymerization [40, 41]. Cells attached to culture surfaces form actin fibers that play important roles in cell survival [42]. hUC-MSC sheets exhibit cytoskeletal F-actin fibers, indicating active actin polymerization even after cell detachment from TRCD (Fig. 3). This suggests that hUC-MSC sheets retain integrin β-1 (for cell–ECM junctions) and F-actin fibers that facilitate cell sheet survival compared to conventional trypsin harvesting treatment. As a result, cell survival rates in harvested hUC-MSC sheets are significantly higher than those for MSCs harvested with chemical disruption methods (Fig. 6). These findings are consistent with the importance of cell–ECM junctions and actin fibers for cell survival.

MSC’s major functional characters in treatment are that (1) differentiation to desired tissue and (2) paracrine factor secretion ability [43]. Both 0.05% trypsin treatment (data not shown) and cell sheet groups (Fig. 2e) showed positive expression of a known stem cell surface marker, CD44. However, cell sheet groups showed significantly higher gene expression level related to paracrine factor secretion ability, compared to 0.05% trypsin treatment for the 5 min group (Fig. 7). Furthermore, in Figs. 3, 4, and 5, the cell sheet group showed tissue-like connected structure of cell adhesion proteins (F-actin, vinculin, fibronectin, laminin, integrin β-1, and connexin 43) in IHC analysis, while 0.05% trypsin treatment for the 5 min group had cleaved cell adhesion protein structures even though both groups showed similar amount of cell adhesion proteins in western blotting data. These findings indicate that the cell sheet having tissue-like connected structure of cell adhesion protein is crucial to MSC’s paracrine actor secretion ability.

Cell sheet technology exhibits higher therapeutic benefits from stem cell therapy in preclinical studies. Furthermore, seven human diseases have been treated using autologous human cell sheets in clinical studies (e.g., heart, cornea, esophagus, periodontal, middle ear, knee cartilage, and lung) [12–18]. This study suggests that the connected tissue-like structure of extracellular and intercellular protein in harvested cell sheets are important to improve cell survival engraftment and therapeutic functions.

## Conclusions

We demonstrate that retention of cellular structures such as ECM, cell–cell junctions, and cell–ECM junctions is correlated with hUC-MSC survival after detachment from cell culture surfaces. Cell sheet technology facilitates cell harvest in sheet form without using any proteolytic enzymes (without chemical disruption). Harvested monolayer hUC-MSC sheets retain structures required for engraftment and tissue renewal, including ECMs, cell–cell junctions, and cell–ECM junctions, correlating with higher cell survival rates and paracrine factor secretion ability post-harvest compared to conventional chemical disruption harvesting methods common to current MSC culture (e.g., trypsin treatments). Preserving the inter-connected tissue-like structure mediated by cell adhesion proteins without any structural disruption is important for effective cell-based therapy.

## Data Availability

The datasets generated and/or analyzed during the current study are not publicly available but are available from the corresponding author on reasonable request.

## Abbreviations

hUC-MSC: Human umbilical cord-derived mesenchymal stem cell
ECM: Extracellular matrix
MSC: Mesenchymal stem cells
YAP: Yes-associated protein
pYAP: Phospo-yes-associated protein
ES cells: Embryonic stem cells
iPS cells: Pluripotent stem cell
EDTA: Ethylenediaminetetraacetic acid
PIPAAm: Poly *N*-isopropylacrylamide
LCST: Lower critical solution temperature
TRCD: Temperature-responsive cell culture dishes
hUC-MSC: Human umbilical cord-derived mesenchymal stem cells
DMEM: Dulbecco’s modified Eagle’s medium
FBS: Fetal bovine serum
NEAA: MEM non-essential amino acids
TCP: Tissue culture polystyrene
PFA: Paraformaldehyde
SEM: Scanning electron microscopy
HMDS: Hexamethyldisilazane
TEM: Transmission electron microscope
PBS: Phosphate-buffered saline
PVDF: Polyvinylidene fluoride or polyvinylidene difluoride
GAPDH: Glyceraldehyde 3-phosphate dehydrogenase
HGF: Hepatocyte growth factor
VEGF: Vascular endothelial growth factor
IL-10: Interleukin 10

## References

[1] Galipeau J, Sensebe L (2018). Mesenchymal stromal cells: clinical challenges and therapeutic opportunities. Cell Stem Cell.

[2] Coelho MB, Cabral JM, Karp JM (2012). Intraoperative stem cell therapy. Annu Rev Biomed Eng.

[3] Ikebe C, Suzuki K (2014). Mesenchymal stem cells for regenerative therapy: optimization of cell preparation protocols. Biomed Res Int.

[4] Brown MA, Wallace CS, Anamelechi CC, Clermont E, Reichert WM, Truskey GA (2007). The use of mild trypsinization conditions in the detachment of endothelial cells to promote subsequent endothelialization on synthetic surfaces. Biomaterials..

[5] Amaral KF, Rogero MM, Fock RA, Borelli P, Gavini G (2007). Cytotoxicity analysis of EDTA and citric acid applied on murine resident macrophages culture. Int Endod J.

[6] Batista U, Garvas M, Nemec M, Schara M, Veranic P, Koklic T (2010). Effects of different detachment procedures on viability, nitroxide reduction kinetics and plasma membrane heterogeneity of V-79 cells. Cell Biol Int.

[7] Shimizu T, Yamato M, Kikuchi A, Okano T (2003). Cell sheet engineering for myocardial tissue reconstruction. Biomaterials..

[8] Akiyama Y, Kikuchi A, Yamato M, Okano T (2004). Ultrathin poly(N-isopropylacrylamide) grafted layer on polystyrene surfaces for cell adhesion:detachment control. Langmuir..

[9] Kato Y, Iwata T, Morikawa S, Yamato M, Okano T, Uchigata Y (2015). Allogeneic transplantation of an adipose-derived stem cell sheet combined with artificial skin accelerates wound healing in a rat wound model of type 2 diabetes and obesity. Diabetes..

[10] Kim SR, Yi HJ, Lee YN, Park JY, Hoffman RM, Okano T, Shim IK, Kim SC (2018). Engineered mesenchymal stem-cell-sheets patches prevents postoperative pancreatic leakage in a rat model. Sci Rep.

[11] Miyahara Y, Nagaya N, Kataoka M, Yanagawa B, Tanaka K, Hao H, Ishino K, Ishida H, Shimizu T, Kangawa K (2006). Monolayered mesenchymal stem cells repair scarred myocardium after myocardial infarction. Nat Med.

[12] Nishida K, Yamato M, Hayashida Y, Watanabe K, Maeda N, Watanabe H, Yamamoto K, Nagai S, Kikuchi A, Tano Y, Okano T (2004). Functional bioengineered corneal epithelial sheet grafts from corneal stem cells expanded ex vivo on a temperature-responsive cell culture surface. Transplantation..

[13] Yamamoto K, Yamato M, Morino T, Sugiyama H, Takagi R, Yaguchi Y, Okano T, Kojima H (2017). Middle ear mucosal regeneration by tissue-engineered cell sheet transplantation. NPJ Regen Med..

[14] Sawa Y, Miyagawa S, Sakaguchi T, Fujita T, Matsuyama A, Saito A, Shimizu T, Okano T (2012). Tissue engineered myoblast sheets improved cardiac function sufficiently to discontinue LVAS in a patient with DCM: report of a case. Surg Today.

[15] Nishida K, Yamato M, Hayashida Y, Watanabe K, Yamamoto K, Adachi E, Nagai S, Kikuchi A, Maeda N, Watanabe H, Okano T, Tano Y (2004). Corneal reconstruction with tissue-engineered cell sheets composed of autologous oral mucosal epithelium. NE J Med.

[16] Kanzaki M, Takagi R, Washio K, Kokubo M, Yamato M (2017). Bio-artificial pleura using an autologous dermal fibroblast sheet. NPJ Regen Med.

[17] Ebihara G, Sato M, Yamato M, Mitani G, Kutsuna T, Nagai T, Ito S, Ukai T, Kobayashi M, Kokubo M (2012). Cartilage repair in transplanted scaffold-free chondrocyte sheets using a minipig model. Biomaterials..

[18] Iwata T, Yamato M, Washio K, Ando T, Okano T, Ishikawa I (2015). Cell sheets for periodontal tissue engineering. Curr Oral Health Rep.

[19] Patel AN, Vargas V, Revello P, Bull DA (2013). Mesenchymal stem cell population isolated from the subepithelial layer of umbilical cord tissue. Cell Transplant.

[20] Riordan NH, Morales I, Fernandez G, Allen N, Fearnot NE, Leckrone ME, Markovich DJ, Mansfield D, Avila D, Patel AN (2018). Clinical feasibility of umbilical cord tissue-derived mesenchymal stem cells in the treatment of multiple sclerosis. J Transl Med.

[21] Hanley PJ, Mei Z, da Graca C-HM, Klis M, Li W, Zhao Y, Durett AG, Zheng X, Wang Y, Gee AP, Horwitz EM (2013). Manufacturing mesenchymal stromal cells for phase I clinical trials. Cytotherapy..

[22] Bartmann C, Rohde E, Schallmoser K, Purstner P, Lanzer G, Linkesch W, Strunk D (2007). Two steps to functional mesenchymal stromal cells for clinical application. Transfusion..

[23] Heng BC, Cowan CM, Basu S (2009). Comparison of enzymatic and non-enzymatic means of dissociating adherent monolayers of mesenchymal stem cells. Biol Procedures Online.

[24] Glenn JD, Whartenby KA (2014). Mesenchymal stem cells: emerging mechanisms of immunomodulation and therapy. World J Stem Cells.

[25] Goodman-Bacon AJ, Nikpay SS (2017). Per capita caps in Medicaid - lessons from the past. N Engl J Med.

[26] Mooney DJ, Vandenburgh H (2008). Cell delivery mechanisms for tissue repair. Cell Stem Cell.

[27] Kocher AA, Schlechta B, Gasparovicova A, Wolner E, Bonaros N, Laufer G (2007). Stem cells and cardiac regeneration. Transpl Int.

[28] Li L, Chen X, Wang WE, Zeng C (2016). How to improve the survival of transplanted mesenchymal stem cell in ischemic heart?. Stem Cells Int.

[29] Besingi RN, Clark PL (2015). Extracellular protease digestion to evaluate membrane protein cell surface localization. Nat Protoc.

[30] Albuschies J, Vogel V (2013). The role of filopodia in the recognition of nanotopographies. Sci Rep.

[31] Sekine H, Shimizu T, Dobashi I, Matsuura K, Hagiwara N, Takahashi M, Kobayashi E, Yamato M, Okano T (2011). Cardiac cell sheet transplantation improves damaged heart function via superior cell survival in comparison with dissociated cell injection. Tissue Eng Part A.

[32] Chang D, Shimizu T, Haraguchi Y, Gao S, Sakaguchi K, Umezu M, Yamato M, Liu Z, Okano T (2015). Time course of cell sheet adhesion to porcine heart tissue after transplantation. PLoS One.

[33] Haraguchi Y, Shimizu T, Yamato M, Kikuchi A, Okano T (2006). Electrical coupling of cardiomyocyte sheets occurs rapidly via functional gap junction formation. Biomaterials..

[34] Boyce M, Yuan J (2006). Cellular response to endoplasmic reticulum stress: a matter of life or death. Cell Death Differ.

[35] Hao J, Zhang Y, Wang Y, Ye R, Qiu J, Zhao Z, Li J (2014). Role of extracellular matrix and YAP/TAZ in cell fate determination. Cell Signal.

[36] Shen B, Delaney MK, Du X (2012). Inside-out, outside-in, and inside-outside-in: G protein signaling in integrin-mediated cell adhesion, spreading, and retraction. Curr Opin Cell Biol.

[37] Kim SH, Turnbull J, Guimond S (2011). Extracellular matrix and cell signalling: the dynamic cooperation of integrin, proteoglycan and growth factor receptor. J Endocrinol.

[38] Huang C, Holfeld J, Schaden W, Orgill D, Ogawa R (2013). Mechanotherapy: revisiting physical therapy and recruiting mechanobiology for a new era in medicine. Trends Mol Med.

[39] Codelia VA, Irvine KD (2012). Hippo signaling goes long range. Cell..

[40] Zhao B, Li L, Wang L, Wang CY, Yu J, Guan KL (2012). Cell detachment activates the Hippo pathway via cytoskeleton reorganization to induce anoikis. Genes Dev.

[41] Elbediwy A, Vincent-Mistiaen ZI, Spencer-Dene B, Stone RK, Boeing S, Wculek SK, Cordero J, Tan EH, Ridgway R, Brunton VG (2016). Integrin signalling regulates YAP and TAZ to control skin homeostasis. Development..

[42] Bachir Alexia I., Horwitz Alan Rick, Nelson W. James, Bianchini Julie M. (2017). Actin-Based Adhesion Modules Mediate Cell Interactions with the Extracellular Matrix and Neighboring Cells. Cold Spring Harbor Perspectives in Biology.

[43] Bergmann A, Steller H (2010). Apoptosis, stem cells, and tissue regeneration. Sci Signal.

